# Management of Severe Traumatic Brain Injury in Pediatric Patients

**DOI:** 10.3389/ftox.2022.910972

**Published:** 2022-06-24

**Authors:** Austin Lui, Kevin K. Kumar, Gerald A. Grant

**Affiliations:** ^1^ Touro University College of Osteopathic Medicine, Vallejo, CA, United States; ^2^ Department of Neurosurgery, Stanford University, Stanford, CA, United States; ^3^ Division of Pediatric Neurosurgery, Lucile Packard Children’s Hospital, Palo Alto, CA, United States; ^4^ Department of Neurosurgery, Duke University, Durham, NC, United States

**Keywords:** traumatic brain injury, pediatrics, molecular pathogenesis, clinical management, algorithm

## Abstract

The optimal management of severe traumatic brain injury (TBI) in the pediatric population has not been well studied. There are a limited number of research articles studying the management of TBI in children. Given the prevalence of severe TBI in the pediatric population, it is crucial to develop a reference TBI management plan for this vulnerable population. In this review, we seek to delineate the differences between severe TBI management in adults and children. Additionally, we also discuss the known molecular pathogenesis of TBI. A better understanding of the pathophysiology of TBI will inform clinical management and development of therapeutics. Finally, we propose a clinical algorithm for the management and treatment of severe TBI in children using published data.

## Introduction

TBI is defined as injury to the head and to the underlying brain caused by an external force, causing an impairment in brain function. According to the Center for Diseases Control and Prevention (CDC), approximately 224,000 people in the United States in 2017 were hospitalized due to TBI, and 7.8% of these people were children ages 0–17 years old (Peterson, 2021). There were around 61,000 deaths related to TBI in the United States in 2017 and around 4.5% of these deaths were in children (Peterson, 2021). TBI also affects the pediatric population globally. For example, in Korea, 31.9% of children that were admitted to hospitals were diagnosed with TBI between June 2008 and May 2009 (Kim et al., 2012). Similarly, it was reported in India that 21% of children admitted to the emergency department in a large teaching hospital were due to TBI (Tabish et al., 2006).

The age and sex of a child also seems to be a factor that influences the likelihood of sustaining a TBI. Several studies report that very young children (0–2 years old) and adolescents (15–18 years old) both are the most likely to experience TBI, while children whose age are in between are less likely to experience TBI by comparison (Langlois and Thomas, 2004; Keenan and Bratton, 2006; Schneier et al., 2006; Amaranath et al., 2014; Greene et al., 2014; Majdan et al., 2014; Dewan et al., 2016). Studies indicate that male children are more likely to be hospitalized or die due to TBI compared to their female counterparts (Langlois and Thomas, 2004; Keenan and Bratton, 2006; Peterson, 2021). However, in the context of athletics, the rate of concussion in girls’ sports are higher than those in boys’ sports (Dick, 2009; Covassin and Elbin, 2011; Lincoln et al., 2011; Marar et al., 2012; Rosenthal et al., 2014; Arambula et al., 2019). While uncertain, possible reasons for difference in concussion rate may be secondary to anatomic differences between sexes, such as head mass and neck girth (Covassin and Elbin, 2011; Arambula et al., 2019). Further research exploring the underlying mechanisms of sex differences in TBI would be of value in guiding treatment recommendations.

Across all ages, the most common causes of TBI are falls, being struck by an object, motor vehicle accident, assault, and self-harm (Control, 2014; Capizzi et al., 2020). According to the CDC data, the most common mechanisms of injury for TBI that result in death are self-harm, falls, and motor vehicle accidents (Control, 2014; Capizzi et al., 2020). The mechanisms of injury for TBI depends also heavily on the region in the world. For example, in Bronx, New York, it was found that 34% of TBIs were due to violence, while 32% of TBI cases were from falls, and 27% from traffic accidents (Cooper et al., 1983; Bruns and Hauser, 2003). However, in France, the majority of TBI cases were caused by traffic accidents (60% of TBI cases) (Tiret et al., 1990; Bruns and Hauser, 2003). In children, the most common causes of TBI are falls and motor vehicle collisions (Dewan et al., 2016). Importantly, international differences in reporting procedures may limit the comparability of data between nations and healthcare systems.

In the United States, around 10% of all cases of TBIs are severe TBI (Wagner, 2016; Capizzi et al., 2020). From the 2006 Kid’s Inpatient Database, which includes data from more than 3,000 hospitals in the United States, it is estimated that 45,875 children were hospitalized due to TBI, while 7,889 children were hospitalized due to severe TBI (Stanley et al., 2012). Severe TBI in children is a large economic burden in the United States, with medical costs and productivity losses costing around $60.4 billion dollars (Corso et al., 2006; Bell et al., 2017).

Given the prevalence of severe TBI in the pediatric population, it is crucial for there to be a guideline TBI management plan particularly for this population. However, despite this need, there are a limited number of research articles studying the management of severe TBI in children. In this review, we seek to delineate the differences between severe TBI management in adults and children. In addition, we summarize the molecular pathogenesis of TBI. Lastly, we propose a clinical algorithm for the management and treatment of severe TBI in children using published data.

## Classification of TBI

Classification of TBI is based on clinical severity or physical causes (Saatman et al., 2008; Hawryluk and Manley, 2015). However, due to the diverse causes of TBI and our limited understanding of the pathophysiology of the different types of TBI, classification of TBI is not as refined and robust as the classification for other diseases (Saatman et al., 2008; Hawryluk and Manley, 2015). Classification by physical mechanism is based on whether the injury is blunt or penetrating (Hawryluk and Manley, 2015). Under classification by physical mechanism, we can also classify TBI as primary or secondary (Hawryluk and Manley, 2015; Capizzi et al., 2020). Primary injury is when a direct hit or indirect hit (acceleration-deceleration mechanism) causes the damage, and secondary injury is related to cellular and molecular damage, vasogenic edema (extracellular edema), and cytogenic edema (intracellular edema) (Elovic and Galang, 2010; Wagner, 2016; Capizzi et al., 2020).

Another method of classifying TBI is through the pathoanatomy (diffuse vs. focal). Focal injury is mostly due to direct contact while diffuse is more likely due to acceleration-deacceleration forces (Gennarelli, 1985; Hawryluk and Manley, 2015). Focal injury can be further broken down into different types: brain contusion, intraparenchymal brain hemorrhage, subdural hematoma, epidural hematoma, and subarachnoid hemorrhage (Andriessen et al., 2010).

Classification of TBI can also be based off severity. According to the CDC, the classification of the severity of TBI depends on the Glasgow Coma Scale (GCS), with mild TBI having a GCS score of 13–15, moderate TBI having a GCS score of 9–12, and severe TBI having a GCS score of lower than 9 (Teasdale and Jennett, 1974; 1976). Classification of TBI can also be based on the loss of consciousness, with 0–30 min of loss of consciousness as mild TBI, between 30 min and 24 h as moderate TBI, and more than 24 h as severe TBI (Defense, 2016; Capizzi et al., 2020).

In addition to these different classifications, there are additional classifications that are not widely utilized including, the Brussel Coma Grades and Innsbruck Coma Scale (Gerstenbrand and Lücking, 1970; Brihaye et al., 1978; Hawryluk and Manley, 2015). There are efforts to develop next generation classification of TBI in a more precise and personalized fashion, where treatment is based off of biomarkers within individual patients (Okonkwo et al., 2013; Hawryluk and Manley, 2015).

## Pathophysiology and Molecular Pathogenesis of TBI

**FIGURE 1:**
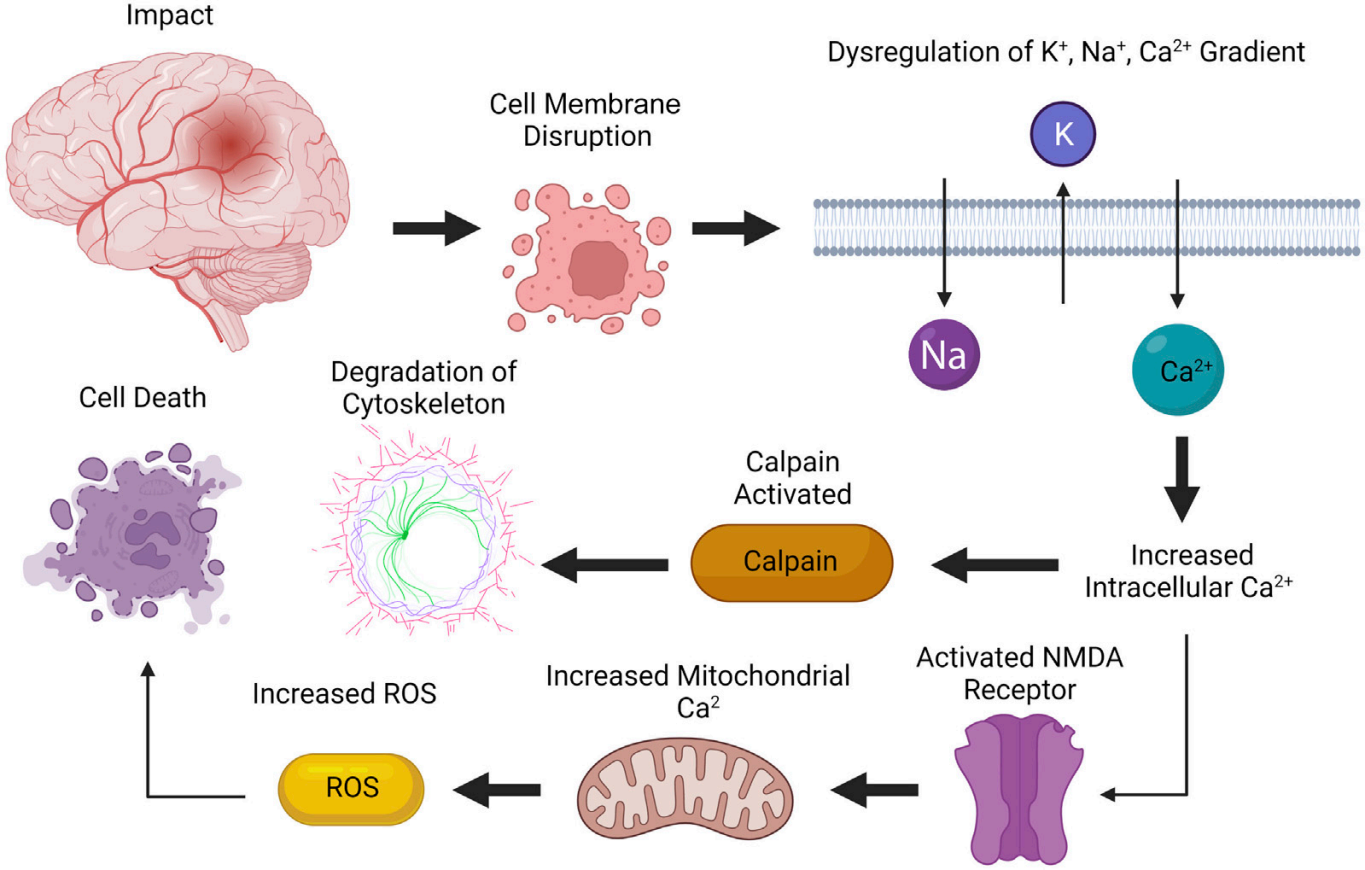
Primary phase of injury from TBI. The primary phase of injury occurs during impact, leading to disruption of cellular membranes. Increased intracellular calcium levels activates calpain, leading to degradation of cytoskeleton. Calcium also activates NMDA receptors, causing increased mitochondrial calcium levels, which increases the concentration of reactive oxygen species (ROS), leading to cell death. Created with BioRender.com.

The molecular pathogenesis of TBI occurs in two phases: an acute primary injury and a delayed secondary injury (Table 1) (Pearn et al., 2017; Sulhan et al., 2020). The primary phase occurs during impact, which leads to the disruption of cellular membranes, resulting in a dysregulation of concentration gradients for potassium, sodium, and calcium (Figure 1) (Wolf et al., 2001; Cuccurullo, 2010; Dixon, 2017; Pearn et al., 2017; Eapen and Cifu, 2018; Capizzi et al., 2020; Sulhan et al., 2020). Increased intracellular calcium levels activates a protease called calpain, leading to the degradation of the cytoskeleton (Pearn et al., 2017; Sulhan et al., 2020). Additionally, calcium also activates N-methyl-D-aspartate (NMDA) receptors, causing cell depolarization and increased mitochondrial calcium levels, which leads to the buildup of reactive oxygen species (ROS), leading to cell death (Pearn et al., 2017). Oxidative metabolism is also altered, which results in lactate production, acidosis, and edema (Pearn et al., 2017; Sulhan et al., 2020).

**TABLE 1:** Differences in TBI pathophysiology between the acute primary phase and the delayed secondary phase of injury.

Characteristics	Acute Primary Phase	Delayed Secondary Phase
Cell membrane destruction	Yes	Yes
Dysregulation of ion gradients	Yes	Yes
Calcium-mediated activation of NMDA receptor	Yes	Yes
Calcium-mediated activation of Calpain	Yes	Yes
Induction of ROS	Yes	Yes
Cell death	Yes	Yes
Reduction of tight junctions	No	Yes
Upregulation of *AQP1* and *AQP4*	No	Yes
BBB breakdown	No	Yes
Influx of immune cells (neutrophils, macrophages, lymphocytes)	No	Yes
Microgliosis and astrogliosis	No	Yes
Glial scar formation	No	Yes
Release of excitatory neurotransmitters	No	Yes
Activation of AMPA receptor	No	Yes
Formation of mPTP	No	Yes
Release of cytochrome C and AIF	No	Yes
Caspase and calcineurin-induced cell death	No	Yes

**FIGURE 2:**
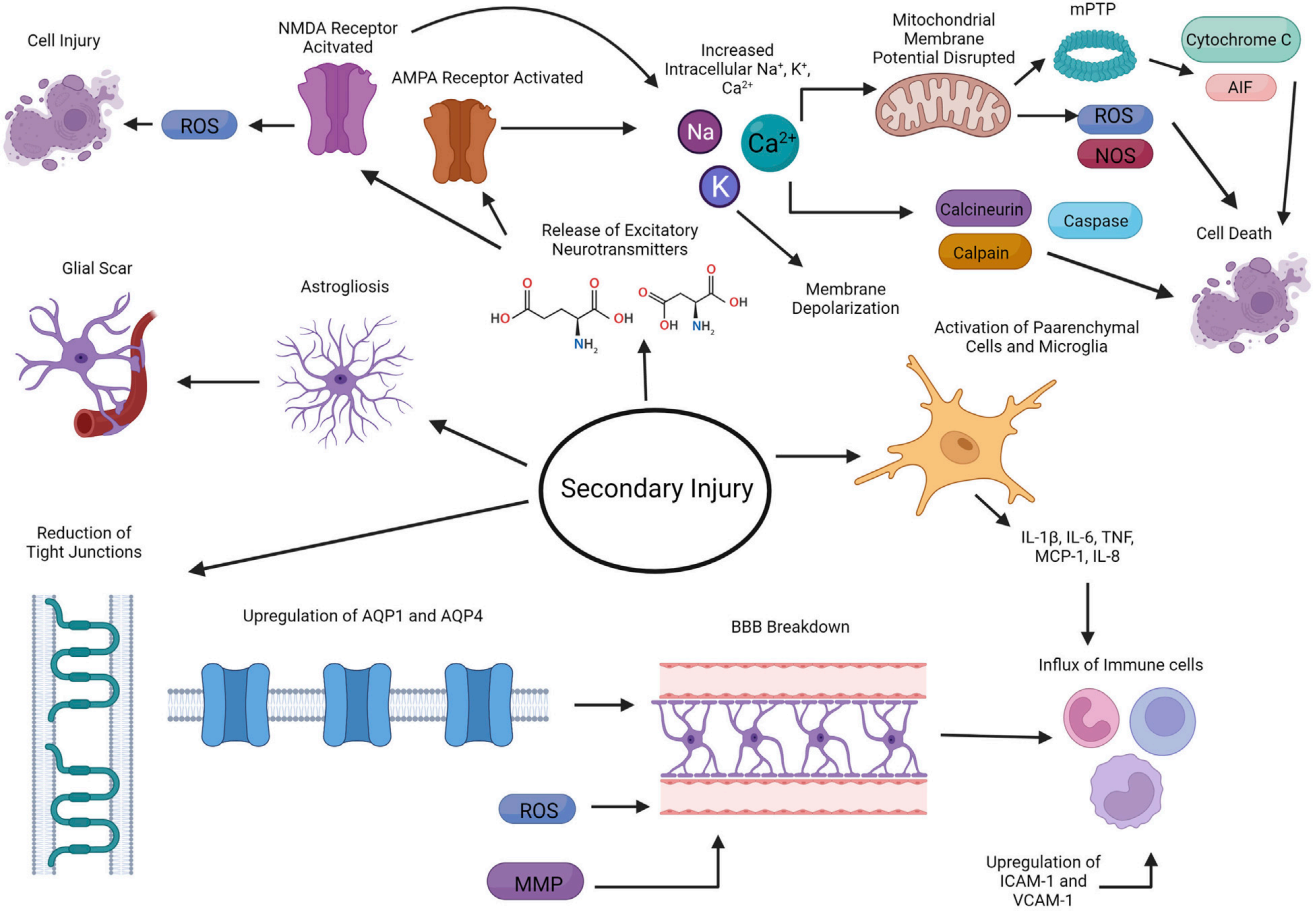
Secondary delayed phase of injury from TBI. The second delayed phase of injury consists of the breakdown of the blood brain barrier (BBB), induction of neuroinflammation, excitotoxity, oxidative stress, apoptosis, and mitochondria dysfunction. Created with BioRender.com.

The second delayed phase consists of the breakdown of the blood brain barrier (BBB), induction of neuroinflammation, excitotoxity, oxidative stress, apoptosis, and mitochondria dysfunction, leading to further damage (Figure 2) (Pearn et al., 2017; Ng and Lee, 2019). Upregulation of *aquaporin 1* (*AQP1*) and *aquaporin 4* (AQP 4) on endothelial cells and reduction of tight junction proteins are factors that causes BBB disruption (Liu et al., 2017; Sulhan et al., 2020). The production of metalloproteinases (MMPs) and ROS also leads to the disruption of the integrity of the BBB (Sulhan et al., 2020). In rodent models, it is found that there is a biphasic increase in BBB permeability (Shapira et al., 1993; Baldwin et al., 1996; Başkaya et al., 1997; Hicks et al., 1997; Chodobski et al., 2011), with the first opening associated with the influx of neutrophils (Chodobski et al., 2011). The mechanism of the second increase in BBB permeability is less well studied (Chodobski et al., 2011).

Neuroinflammation also plays an important role in pathophysiology of TBI. Parenchymal cells along with microglia in the brain start producing proinflammatory cytokines such as IL-1β, IL-6 and TNF, which leads to the influx of inflammatory cells into the brain, including neutrophils, monocytes/macrophages, and lymphocytes (Ahn et al., 2004; Buttram et al., 2007; Goodman et al., 2008; Lotocki et al., 2009; Frugier et al., 2010; Semple et al., 2010; Chodobski et al., 2011; Aungst et al., 2014; Pearn et al., 2017; Ng and Lee, 2019). Chemokines, including MCP-1 and IL-8, are also upregulated in the injured tissue, which further helps recruit other leukocytes to the site of injury (Kossmann et al., 1997; Buttram et al., 2007; Bye et al., 2007; Semple et al., 2010; Ng and Lee, 2019). An increase in expression of VCAM-1 and ICAM-1 on endothelial cells and leukocytes/endothelial cells, respectively, facilitates the recruitment of leukocytes to site of injury (Carlos et al., 1997; Rancan et al., 2001; Ng and Lee, 2019). Astrogliosis and microgliosis also occurs, further contributing to the neuroinflammation occurring at the site injury (Chiu et al., 2016). Ultimately, astrocytes then form a glia scar, which serves as a barrier to separate the damaged tissue from healthy tissue (Fitch and Silver, 2008). Although neuroinflammation is crucial to the repair and recovery from TBI, chronic and persistent neuroinflammation is thought to also contribute to neurodegeneration (Simon et al., 2017).

In addition to neuroinflammation, TBI also induces the release of excitatory neurotransmitters such as glutamate and aspartate from neurons (Chamoun et al., 2010). These neurotransmitters can then bind to NMDA and α-amino-3-hydroxy-5-methyl-4-isoxazolepropionic acid receptor (AMPA) receptors, leading to the influx of sodium, calcium and potassium, which subsequently depolarizes these neurons (Ng and Lee, 2019). Calcium in particular can activate calcineurin, calpain, and caspases, which leads to apoptosis while activation of NMDA ultimately leads to the production of ROS, further exacerbating neural injury (Ng and Lee, 2019).

Mitochondria dysfunction is another consequence of TBI. Increases in calcium concentrations are buffered by the mitochondria *via* a variety of transporters, including the mitochondrial calcium uniporter and uncoupling proteins (Williams et al., 2013). This buffering can lead to disruption of the mitochondria membrane potential, which drives further increase in ROS and reactive nitrogen species (RNS) (Lamade et al., 2020). Upon loss of mitochondria membrane potential, the mitochondrial permeability transition pore (mPTP) formation begins (Szabó and Zoratti, 1991). The formation of mPTP allows for the release of proteins including cytochrome c and apoptosis-inducing factor (AIF), leading to both caspase-dependent and independent cell death (Lamade et al., 2020).

## TBI in Pediatric Population Versus Adult Population

There exist major physiological and anatomical differences between adults and children which impact management of TBI (Supplementary Table S1). Since the fontanelles and sutures may be open in very young pediatric patients, it is possible that these patients can mitigate the rise in intracranial pressure (ICP) levels (Figaji, 2017). The opening of the fontanelles and sutures increase cerebral compliance in young children (Figaji, 2017). It should also be noted that BP and ICP are generally lower in pediatric patients, and even a small increase can lead to significant adverse events (Figaji, 2017). If autoregulation is impaired in a pediatric patient, then high BP can be harmful (Figaji, 2017). While there are recommended values for cerebral perfusion pressure (CPP) in children, the evidence for these values are not well supported (Figaji, 2017).

Children also have smaller lung volumes, which must be taken into consideration when managing ventilatory status. Caution must be taken to not hyperventilate the child, which can lead to hypocapnia and ischemia (Figaji, 2017). The airway of a pediatric patient is also more prone to obstruction (Figaji, 2017). While some guidelines, such as the 2019 Management of Pediatric Severe Traumatic Brain Injury by Kochanek et al. (2019b) include hypothermia as a second-tier therapy, there is little to no evidence showing the effectiveness of this type of therapy in children (Hutchison et al., 2008; Adelson et al., 2013; Crompton et al., 2017; Figaji, 2017; Lewis et al., 2017). However, there is evidence supporting the prevention of hyperthermia in children (Figaji, 2017). There are also no standardized formulations for hyperosmolar therapy for children (Kochanek et al., 2012; Bell et al., 2013; Figaji, 2017). Finally, the use of decompressive craniectomy is seen as controversial (Taylor et al., 2001; Figaji et al., 2003; Figaji et al., 2006; Kan et al., 2006; Rutigliano et al., 2006; Jagannathan et al., 2007; Figaji et al., 2008; Adamo et al., 2009; Appelboom et al., 2011; Pérez Suárez et al., 2011; Figaji, 2017).

## Management of TBI in Adults Versus Children

### Neuroimaging

During the initial management of severe TBI (GCS 8 or less), both adults and children should undergo a computerized tomography (CT) scan (Wintermark et al., 2015). In addition to initial head CT, children also require additional imaging of the cervical spine and head along with radiographs and focused assessment with sonography for trauma (FAST). Cervical spine imaging in pediatric TBI is recommended secondary to the high association of injury to posterior ligamentous complex in abusive head trauma and accidental TBI (Choudhary et al., 2014; Baerg et al., 2017; Oh et al., 2017; Henry et al., 2021). Since many TBI pediatric cases may also involve trauma to other areas of the body, using FAST to detect trauma to the abdominal region is recommended. In children, it is important to not exclude the possibility of elevated ICP, despite a normal initial CT scan (Bailey et al., 2012; Kochanek et al., 2019b). Given that CT imaging findings may not detect intracranial hypertension, serial CT scans have limited value in guiding ongoing treatment particularly in the case of mild TBI (Tabori et al., 2000; Figg et al., 2006; da Silva et al., 2008; Rangel-Castilla et al., 2008; Bata and Yung, 2014). It is also not recommended to repeat CT scans performed more than 24 h after admission in severe TBI pediatric patients, unless ICP is increased or if patient has neurologic deterioration (Kochanek et al., 2019b). In contrast, adults with severe TBI may benefit from repeat head CT, as more evident radiographic changes can inform clinical management (Brown et al., 2004; Wang et al., 2006; Thomas et al., 2010).

### ICP Management

Initial management of ICP includes elevating the head at 30° while maintaining the neck in a neutral position (Marehbian et al., 2017). In adults, it is recommended that the ICP is kept at 22 mmHg or lower (Carney et al., 2017; Schizodimos et al., 2020), because values above 22 mmHg are associated with increased levels of mortality (Carney et al., 2017). In children, it is suggested that the ICP should be kept lower than 20 mmHg (Kochanek et al., 2019b), since ICP higher than 20 mmHg is associated with poor clinical outcomes, including lower survival rates and disability (Esparza et al., 1985; Michaud et al., 1992; Jagannathan et al., 2008; Kukreti et al., 2014). Infusion of hypertonic saline (3% saline 0.1–1.0 ml/kg of body weight per hour) in children is recommended to keep ICP lower than 20 mmHg (Kochanek et al., 2019b). It is recommended that children with severe TBI (Kochanek et al., 2019b) and adults with GCS 3-8 and abnormal CT scan results (hematomas, contusions, swelling, herniation, or compressed basal cisterns) should have their ICP monitored (Carney et al., 2017). Additionally, the use of ICP monitoring is also warranted in adults with severe TBI even if they have a normal CT scan, given that two of the following are true: patient is older than 40 years old, patient has unilateral or bilateral motor posturing, or if patient’s systolic blood pressure is less than 90 mmHg (Carney et al., 2017).

### CSF Drainage

Cerebrospinal fluid (CSF) drainage is used to lower ICP in both adults and children (Kochanek et al., 2019b). Both lumbar and ventricular drains seem to be effective in lowering ICP (Tuettenberg et al., 2009; Chau et al., 2019). Continuous CSF drainage is preferred over intermittent drainage due to it being more effective and having better control of ICP (Carney et al., 2017). Guidelines suggest that CSF drainage should be used when patient’s GCS is less than 6 during the first 12 h post-injury (Carney et al., 2017). Attempts to analyze the optimal timing of CSF diversion were limited due to high levels of bias and heterogeneity among published data (Chau et al., 2020). However, the majority of studies identified in this report simultaneously placed ICP monitors and ventricular drains or utilize a ventricular drain for both ICP monitoring and subsequent CSF drainage (Chau et al., 2020).

### Fluids

Isotonic saline can be used to maintain euvolemia (Myburgh et al., 2007). In children, more than 2 h of serum sodium levels above 170 mEq/L should be avoided due to complications of anemia and thrombocytopenia (Kochanek et al., 2019b). Sustained serum sodium levels above 160 mEq/L should also be avoided since it can cause complications related to deep vein thrombosis (Kochanek et al., 2019b). Notably, serum osmolarity of greater than 320 mOsm/L can result in neurological and renal side-effects (Bullock, 1995).

### Blood Pressure

It is recommended that systolic blood pressure be maintained at or above 100 mmHg for patients 50–69 years old, and at or above 110 mmHg for patients between 15 and 49 years old and patients above 70 years old (Carney et al., 2017). The blood pressure needed to maintain minimum CPP allowing for metabolic needs to be met in children with severe TBI is not known, but largely depends on the age of the child (Figaji, 2017). Nonetheless, systolic blood pressure should be above the fifth percentile for the child’s age, since higher systolic blood pressure is thought to improve clinical outcomes (Samant et al., 2008; Suttipongkaset et al., 2018).

### Endotracheal Intubation

Endotracheal intubation is needed to keep the patient’s PaO2 above 60 mmHg (Dellazizzo et al., 2018). In children with major trauma, the use of cuffed endotracheal tubes is recommended (Kleinman et al., 2010). However, complication rates from using cuffed endotracheal tubes in children is similar to that of uncuffed endotracheal tubes in critically ill children (Deakers et al., 1994; Khine et al., 1997).

### Ventilation

Patients should maintain a PaO2 of more than 60 mmHg (Bratton et al., 2007) while maintaining PaCO2 more than 30 mmHg is recommended using end-tidal CO2 to measure CO2 levels (Stocchetti et al., 2005; Bratton et al., 2007). Lowering PaCO2 to less than 30 *via* hyperventilation is sometimes acceptable as a temporary way to resolve an ICP crisis (Diringer et al., 2000). However, it is not recommended to prolong hyperventilation such that it lowers PaCO2 to 25 mmHg or less and when using hyperventilation, measurements of BtpO2 should be used (Carney et al., 2017). It is also not recommended to use hyperventilation during the first 24 h after TBI, which is when cerebral blood flow is reduced (Carney et al., 2017). In children, PaCO2 should be maintained between 35 and 40 mmHg (Sharp and Tieves, 2015), and it is not recommended to induce hyperventilation so that PaCO2 is lower than 30 mmHg during the first 48 h after TBI (Kochanek et al., 2019b). However, if hyperventilation is needed to treat refractory intracranial hypertension, it is suggested that neuromonitoring is used to detect cerebral ischemia (Kochanek et al., 2019b).

### Antiseizure Medications

Antiseizure pharmacological agents are recommended to prevent post-traumatic seizures (PTS) after TBI (Carney et al., 2017). Levetiracetam can be used, although there are insufficient data to say whether levetiracetam or phenytoin is better in early PTS (Carney et al., 2017; Kochanek et al., 2019b). In general, for preventing late PTS, phenytoin or valproate are not recommended while phenytoin can be used for preventing early PTS (Carney et al., 2017). Phenytoin has been shown to significantly reduce seizures during the first week after severe TBI, but this anti-seizure effect is lost after longer periods of time (Temkin et al., 1990).

### Antifibrinolytic Therapy

A loading dose of 1 g of tranexamic acid can be infused over a time span of 10 min, and then an additional infusion of 1 g over 8 h afterwards (Slomski, 2019). Currently, it is unclear if there are benefits of administering tranexamic acid for severe TBI with intracranial hemorrhage (Bossers et al., 2021).

### Venous Thromboembolism Prophylaxis

In the general population, intermittent pneumatic compression should be used when the patient is admitted to the hospital (Abdel-Aziz et al., 2015). In the adult population, low molecular weight heparin (LMWH), enoxaparin, or low-dose unfractionated heparin can be used in combination with intermittent pneumatic compression (Carney et al., 2017). However, with the use of these drugs, there is an increased risk of worsening of intracranial hemorrhage (Carney et al., 2017). Therefore, the clinician should make the judgement to use them based on imaging results and stability of the TBI (Carney et al., 2017). While there is limited data on venous thromboembolism prophylaxis in the pediatric TBI population, there is evidence that LMWH prophylaxis is more effective than unfractionated heparin in preventing venous thromboembolism (van Erp et al., 2021).

### Management of Coagulopathy

There is a variation in what is considered a low platelet count in TBI patients. Current evidence suggests the maintenance of platelet count of patients at above 100,000–175,000/μl, using platelet transfusions if platelet necessary (Joseph et al., 2014). While this recommendation is based on studies in the adult TBI population, there are also several studies demonstrating the unfavorable impact of coagulopathy in pediatric TBI (Kamal et al., 2011; Podolsky-Gondim et al., 2018). In the pediatric TBI population, trauma-related coagulopathy (platelet count < 150,000/μl, fibrinogen < 180 mg/dl, aPTT > 1.2, or prothrombin time > 1.3) in TBI pediatric patients was associated with worse neurological outcomes (Podolsky-Gondim et al., 2018).

### Glucose Management

In severe TBI patients, studies recommended that blood glucose levels are in the range of 140–180 mg/dl, since hyperglycemia or hypoglycemia should be avoided (Sharma and Vavilala, 2012). Increased complications and mortality rates have been associated in TBI patients with hyperglycemia (Alvis-Miranda et al., 2014). However, the SHINE trial demonstrated that stroke patients with an average glucose level of 118 mg/dl did not have significantly more favorable functional outcomes compared to stroke patients with an average glucose level of 179 mg/dl (Johnston et al., 2019). Given these results, the benefit of strict glucose control in children is unclear.

### Temperature Management

In both adults and children, normothermia should be maintained (Carney et al., 2017; Kochanek et al., 2019b). Fever should be prevented by using antipyretic medications, cooling blankets, endovascular temperature management catheters, etc. (Puccio et al., 2009). Deliberate deep hypothermia should not be used to improve outcomes in adult patients (Carney et al., 2017; Kochanek et al., 2019b). In children, protocols recommending moderate hypothermia (32°C–33 °C) for the control refractory intracranial hypertension exist, but there is limited evidence of its efficacy on overall outcomes (Kochanek et al., 2019b).

### Sedation and Analgesia

Patients with ICP elevation should be given a sedative agent and opioids (Rajajee et al., 2017). Fentanyl generally has greater efficacy compared to morphine (Wenderoth et al., 2013). Since propofol is effective in reducing cerebral metabolism and ICP, with a short duration permitting intermittent neurological assessments, it is the general preferred sedative agent (Farling et al., 1989; Pinaud et al., 1990; Flower and Hellings, 2012). However, it is important to also note that propofol may not improve mortality (Carney et al., 2017). Barbiturates are not recommended for prevention of intracranial hypertension (Carney et al., 2017). In children, etomidate is recommended for sedation as it can reduce ICP and improve CPP (Bramwell et al., 2006). If etomidate is not available, then ketamine has been proposed as an alternative (Scherzer et al., 2012). However, several studies demonstrated that ketamine has been associated with increase in ICP, while others have demonstrated its efficacy in decreasing ICP in the pediatric population, therefore making its benefit uncertain (Gibbs, 1972; Wyte et al., 1972; Yoram Ben and Watemberg, 2006; Bar-Joseph et al., 2009; Ketharanathan et al., 2017). Prolonged use of propofol may increase levels of lactic acid and therefore, it is not recommended for usage beyond rapid sequence induction (Flower and Hellings, 2012). The FDA does not recommend the prolonged use of propofol for sedation or refractory intracranial hypertension (Kochanek et al., 2019b). Other agents that can be used are succinylcholine, which is useful when there are airway complications and has greater safety than rocuronium (Hardcastle et al., 2014). Finally, it is recommended to avoid using midazolam and/or fentanyl during ICP crises because of the risk of cerebral hypoperfusion (Kochanek et al., 2019b). Another important consideration regarding long-term administration of sedatives and analgesic drugs is the risk of physiological dependence in pediatric patients (Tobias, 2000; Ista et al., 2007). Multiple studies have demonstrated that those who experience TBI during childhood and/or adolescence are more vulnerable to substance abuse disorders (Corrigan et al., 2013; Ilie et al., 2016; Cannella et al., 2019). Given this risk of dependence, clinicians should consider expeditious weaning of analgesic agents after the acute phase of injury.

### Nutrition

In adults, it is recommended to use transgastric jejunal feeding (to reduce risk of ventilator-associated pneumonia) at least on the 5th day after TBI and at most on the 7th day after TBI (Carney et al., 2017). In children, it is recommended to start nutritional support within 72 h of TBI, as it is shown to approve outcomes (Kochanek et al., 2019b). It is not suggested to use immune-modulating diet in children (Kochanek et al., 2019b).

### Infection Prophylaxis

Although early tracheostomy is used to reduce the number of days the patient is on mechanical ventilation (provided that the benefits outweigh the risk of complications), there is no evidence that early tracheostomy reduces rate of pneumonia or mortality (Carney et al., 2017). Reducing ventilator-associated pneumonia is not recommended since it increases risk of acute respiratory distress syndrome (Carney et al., 2017). Lastly, antimicrobial-impregnated catheters can be used during external ventricular drainage as a prevention against infections (Carney et al., 2017).

### Corticosteroids

In both adults and children, the use of corticosteroids is not recommended method reduce ICP or improve outcomes (Carney et al., 2017; Kochanek et al., 2019b).

### Emergent Pathway if Clinical Evidence of Rapid Neurological Deterioration

In the first days after TBI, the patient is closely monitored in the intensive care unit with multimodal monitoring. If signs or symptoms of cerebral herniation appears even without ICP elevation, such as pupillary dilation, hypertension/bradycardia, and extensor posturing (Kochanek et al., 2019a), then the following should occur: endotracheal intubation, elevation of head to 30–45°, brief hyperventilation targeting a paCO2 of 30 mmHg (Stevens et al., 2015), administration of 1–1.5 g/kg mannitol (Qureshi et al., 2000) or 23.4% sodium chloride 30–60 ml over 10 min (Koenig et al., 2008), and maintenance of mean arterial pressure (MAP) to between 80 and 100 mmHg. In children, we recommend giving 0.5–1 g/kg mannitol or hypertonic saline (1–3 ml/kg up to a maximum of 250 ml for 3% saline or 0.5 ml/kg up to a maximum of 30 ml for 23.4% saline) over 10 min (Kochanek et al., 2019a). It is recommended in children that a bolus of 3% hypertonic saline is given when there is intracranial hypertension, with the effective doses for acute use ranging between 2 and 5 ml/kg over 10–20 min (Kochanek et al., 2019b). After these steps have occurred, an emergency CT scan should be obtained to determine if emergent surgery or a ventriculostomy at the bedside is necessary (Kochanek et al., 2019a).

### ICP Pathway

If the ICP of the patient is raised greater than 20 mmHg (child) or 22 mmHg (adult) for at least 5 min, then drainage of CSF should occur (Kochanek et al., 2019a). If this is not resolved, then patient should receive a bolus and/or infusion of hypertonic saline or mannitol (Kochanek et al., 2019a). For hypertonic saline, administer 3% NaCl to reach sodium concentration of 145–155 mEq/L (Qureshi et al., 1998). Also administer a bolus of 30 ml of 23.4% NaCl if there are acute ICP elevations (Ware et al., 2005). Alternatively, 0.25–1 g/kg bolus of mannitol can also be given every 4–6 h as needed (Carney et al., 2017). It is recommended that patients receiving mannitol should have their serum osmolality maintained below 320 mmol/L (Dinsmore, 2013). It is also important to prevent arterial hypotension (Carney et al., 2017). The use of mannitol should be restricted to patients who, prior to ICP monitoring, have signs of transtentorial herniation or progressive neurological deterioration that is not due to extracranial causes (Carney et al., 2017). It is recommended in children that a bolus of 3% hypertonic saline is given when there is intracranial hypertension, with the effective doses for acute use ranging between 2 and 5 ml/kg over 10–20 min (Kochanek et al., 2019b). For refractory ICP, the use of additional analgesia/sedation is recommended (Kochanek et al., 2019a). If ICP is still above threshold, then neuromuscular blockade is needed, which should be monitored with EEG (Kochanek et al., 2019a). The next line of therapy is to administer additional hypertonic saline/hyperosmolar therapy (Kochanek et al., 2019a). If the elevated ICP is still not resolved, then a CT scan should be repeated to see if hemicraniectomy is required (Kochanek et al., 2019a). As ICP improves along with clinical neurological recovery, then the patient can be weaned off ICP, CCP, and/or PrO2 therapy (Kochanek et al., 2019a).

### CPP Pathway

In adults, a CPP of 60–70 mmHg is recommended (Carney et al., 2017). If the CPP of an adult is below optimal levels and if autoregulation is impaired, it is recommended for the ICP to be lowered instead of elevating mean arterial pressure (Howells et al., 2005). Whether the minimal CPP should be 60 or 70 mmHg depends on the autoregulatory status of the patient (Carney et al., 2017). Due to the risk of respiratory failure in adults, fluids and pressors can be used to maintain a CPP of 70 mmHg (Carney et al., 2017). CPP should be in between 40 and 50 mmHg in children who are 5 years old and under (Kochanek et al., 2019b). Those who are between 5 and 17 years old should have their CPP maintained above 50 mmHg (Stevens et al., 2015). If CPP decreases, the status of the intravascular volume should be checked, and vasopressor infusion and hypertonic saline bolus should be given (Kochanek et al., 2019a). However, if CPP still decreases, then repeat a CT scan to see if surgery should be considered (Kochanek et al., 2019a).

### PbrO2 Pathway

Brain tissue oxygen (PbrO2) should be kept greater than 10 mmHg if PbrO2 monitor is being used. Note that some literature suggests that PbrO2 of less than 20 mmHg is considered compromised and are associated with worse outcomes (van Santbrink et al., 1996; Bardt et al., 1998; Valadka et al., 1998; van den Brink et al., 2000; Chang et al., 2009; Nangunoori et al., 2012). If PbrO2 decreases, then raise the fraction of inspired oxygen (FiO2) on the ventilator. If PbrO2 still lowers, then give vasopressor infusion, adjust PaCO2, and optimize hemoglobin levels (Kochanek et al., 2019a). If this fails, then repeat a imaging for surgical evaluation should be considered.

### Second Tier Therapies

If the patient progresses to impending herniation after the ICP, CPP, and/or PrbO2 pathway, then second tier therapies should be considered. If the repeat imaging reveals a new or growing swelling or hemorrhage, then patient should undergo evacuation and decompressive craniectomy (DC) (Kochanek et al., 2019a). Bifrontal DC is not suggested in patients who have diffuse injury (without having mass lesions) and patients who have ICP elevation of greater than 20 mmHg for more than 15 min within 1 h that are refractory to first-tier therapies (Carney et al., 2017). Also, a large frontotemporoparietal (at or greater than 12 × 15 cm or 15 cm diameter) DC is suggested over a small frontotemporoparietal DC (Carney et al., 2017). It is also appropriate to administer a dose of hypertonic saline or mannitol (Kochanek et al., 2019a). It is better to avoid sodium concentrations greater than 160 mEq/L and osmolarity of greater than 360 mOsm/L (Kochanek et al., 2019a). If hyperosmolar therapy and propofol infusion (titrated to deep sedation) does not lower ICP, then consider putting patient into barbiturate coma (Eisenberg et al., 1988; Vella et al., 2017). Moderate hypothermia (between 32 and 34°) and hyperventilation can also be considered, but this has unclear benefit on overall clinical outcomes (Kochanek et al., 2019a). Advanced neuromonitoring is also recommended, such as brain tissue oxygen monitoring to help guide bedside management of ICP.

## Recovery From TBI and Long-Term Outcomes

There exists difficulty identifying the outcomes that are specifically related to TBI, since trauma that causes TBI may cause multiorgan damage (Stocchetti and Zanier, 2016). However, long-term consequences of TBI include increased likelihood of mortality, vegetative status, physical disabilities, cognitive impairment, psychiatric disorders, seizures, and unemployment rate (Stocchetti and Zanier, 2016). Younger age was associated with higher likelihood of survival at follow-up in an adult trauma center patient cohort (Schroeppel et al., 2020). Based on a retrospective study on TBI outcomes in children, it was found that children under 2 years old who experienced TBI were more likely to experience vomiting and post-traumatic epilepsy as the most common symptom (Hwang et al., 2019). For children above 2 years old, the major long-term symptoms were confusion and disorientation (Hwang et al., 2019). Despite this, children were overall found to improve in functional ability after ongoing sessions of rehabilitation, which shows the resilience in children regarding recovery (Hwang et al., 2019). Overall, physical rehabilitation is helpful in children who suffered from TBI to regain back functionality. It is also found that children who had higher functional status coming into rehabilitation and shorter time between TBI and rehabilitation tend to have higher functionality during discharge and a shorter stay at rehabilitation (Rice et al., 2005). Pediatric rehabilitation not only involves physical rehabilitation, but it also involves an interdisciplinary approach where the child also works with physical and occupational therapists, psychologists, speech therapists, and allied services (Yen and Wong, 2007). This helps the child face life challenges again and gain an enriched experience, which is related to increased neural plasticity (Yen and Wong, 2007).

## Discussion

**FIGURE 3:**
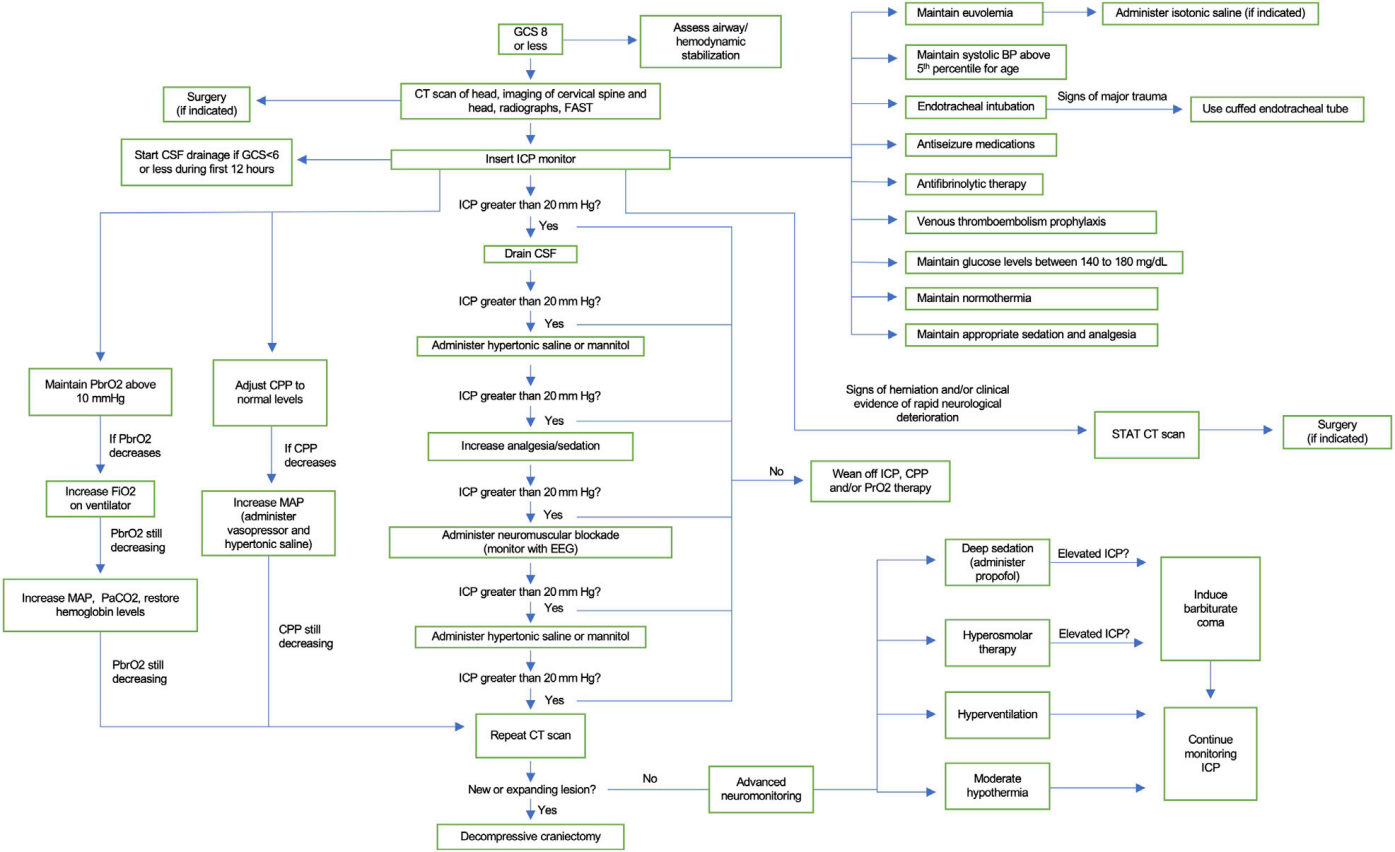
Clinical algorithm for the treatment of severe TBI in children. Baseline care includes neurological examination and assessment of airway and hemodynamic stability. Guidelines for ICP monitoring, volume status, blood pressure, endotracheal intubation, glucose management, temperature, sedation, analgesia, antiseizure medications, antifibrinolytic therapy, and venous thromboembolism prophylaxis are detailed. Uncontrolled ICP, PrbO2, or CCP will lead to a repeat imaging. If a new expanding lesion is found, then decompressive craniectomy is indicated. If negative, second tier therapies can be used, including deep sedation, hyperosmolar therapy, hyperventilation, and moderate hypothermia.

In this review, we summarized the data regarding the epidemiology, classification, molecular pathogenesis, and management of TBI in children and adults. The known pathological mechanisms that lead to decreased functioning in animal models and humans after TBI include neuroinflammation, excitotoxity, oxidative stress, apoptosis, and mitochondria dysfunction. However, future studies are needed to further elucidate the pathophysiology of pediatric TBI so that more effective clinical management and therapeutics can be developed. Despite the anatomical and physiological differences between adults and pediatric populations, there is a limited amount of literature comparing the management of TBI between these two patient groups. There is a paucity of clinical studies on TBI in children compared to the adult literature. As a result, there are unclear evidence-based guidelines for parameters such as CPP and ICP. The majority of TBI guidelines for pediatric patients are adapted from the management of TBI in adults, which may be inappropriate owing to the inherent physiological and anatomical difference between these two demographics. Here we sought to highlight the differences in management of TBI between children and adults and create a clinical management algorithm (Figure 3), which builds on the recent published guidelines from Kochanek et al. (2019). Finally, we also discussed the long-term recovery and outcomes in these patients. Future prospective clinical trials dedicated to studying imaging, intensive care unit (ICU) management, and neurosurgical intervention in the pediatric population would be of tremendous value.
