# Supplementary material for: Management of Severe Traumatic Brain Injury in Pediatric Patients
Source: Front Toxicol. 2022 Jun 24;4:910972. doi: 10.3389/ftox.2022.910972 (PMC9263560; doi:10.3389/ftox.2022.910972)
Supplement: Supplementary file 1 [file Table1.DOCX]

**Severe TBI in adult versus pediatric patients**

| **Parameters** | **Adult** | **Pediatric** | **Shared** |
| --- | --- | --- | --- |
| Neuroimaging |  | Additional imaging of cervical spine and head, radiographs, and FAST scan | Head CT |
| ICP Management | ICP **≤** 22 mmHg | ICP **≤** 20 mmHg | Elevate head to 30 degrees, keep neck in neutral position |
| CSF Diversion |  |  | Continuous CSF drainage preferred over intermittent drainage. Use when GCS is less than 6 hours during first 12 hours post-injury. |
| Endotracheal Intubation |  | If major trauma, use cuffed endotracheal tubes | Keep PaO2 above 60 mmHg |
| Fluids |  |  | Use isotonic saline to maintain euvolemia |
| Blood Pressure | SBP> 100mmHg for patients between 50 to 69 years old  SBP>110 mmHg for patients between 15 to 49 years old and above 70 years old | Optimal blood pressure needed to maintain minimum cerebral perfusion not established  SBP>5^th^ percentile for child’s age |  |
| Ventilation | PaCO2>30 mmHg  Decreasing PaCO2<30 via hyperventilation as a temporary measure during ICP crisis  Prolonged hyperventilation is not recommended with PaCO2<25 mmHg  Not recommended to use hyperventilation during the first 24 hours after TBI | PaCO2 goal 35-40mmHg  Not recommended to induce hyperventilation with PaCO2<30 mmHg during the first 48 hours after TBI | PaO2>60 mmHg |
| Antiseizure Agents |  |  | Antiseizure drugs use is recommended to prevent PTS after TBI  Insufficient data regarding levetiracetam versus phenytoin in early PTS  For preventing late PTS, phenytoin or valproate are not recommended while phenytoin can be used for preventing early PTS |
| Antifibrinolytic Therapy |  |  | 1 g of tranexamic acid infused over 10 minutes, and then infusion of 1 g over 8 hours |
| Venous Thromboembolism Prophylaxis |  |  | Low molecular weight heparin, enoxaparin, or low-dose unfractionated heparin can be used in combination with intermittent pneumatic compression  However, there is increased risk of worsening of intracranial hemorrhage |
| Management of coagulopathy |  |  | Maintenance of the platelet count above 100,000 – 175,000/microL, using platelet transfusions if platelet levels are low |
| Glucose Management |  | Unclear whether there is benefit from strict glucose control | Goal blood glucose levels 140-180 mg/dL |
| Temperature Management | Deliberate deep hypothermia should not be used to improve outcomes | Moderate hypothermia (32◦C to 33◦C) can be used to control ICP levels | Normothermia |
| Sedation and Analgesia | Recommend use of fentanyl and propofol  Barbiturates not recommended for prevention of intracranial hypertension | Etomidate is recommended for sedation, while thiopental and propofol may worsen patients who are hemodynamically unstable.  Ketamine may be an alternative, although unclear benefits  Prolonged use of propofol is not recommended for usage beyond rapid sequence induction  Succinylcholine could be useful when there are airway complications, and with greater safety than rocuronium  It is recommended to avoid using midazolam and/or fentanyl, during ICP crises |  |
| Nutrition | Use transgastric jejunal feeding at least on the 5^th^ day after TBI | Start nutritional support within 72 hours of TBI  Not suggested to use immune-modulating diet in children |  |
| Infection Prophylaxis |  |  | No evidence that early tracheostomy reduces rate of pneumonia or mortality  Antimicrobial-impregnated catheters can be used during external ventricular drainage |
| Corticosteroids |  |  | Use of corticosteroids is not recommended to reduce ICP or improve outcomes |
| Emergent pathway  (for rapid neurological deterioration) | 1 to 1.5 g/kg mannitol or 23.4% sodium chloride 30 to 60 mL should be administered over 10 minutes, and maintenance of mean arterial pressure to between 80 and 100 mmHg | Give 0.5-1 g/kg mannitol or hypertonic saline (1 to 3 ml/kg up to a maximum of 250 ml for 3% saline or 0.5 ml/kg up to a maximum of 30 ml for 23.4% saline) over 10 minutes    3% NaCl should be given when there is intracranial hypertension, with the effective doses for acute use ranging from 2-5 mL/kg over 10-20 minutes | Monitor patient closely with multimodal monitoring  For signs of cerebral herniation: endotracheal intubation, elevation of head, brief hyperventilation to goal paCO2 of 30 mmHg  Head CT scan should be obtained to determine if emergent surgery or a ventriculostomy is necessary |
| ICP Pathway | If ICP >22 mmHg for at least 5 minutes, then drain CSF  If ICP>22 mmHg persistently, administer bolus and/or infusion of hypertonic saline or mannitol.  For hypertonic saline, administer 3% NaCl to reach sodium concentration of 145-155 mEq/L. Also administer a bolus of 30 mL of 23.4% NaCl if there are acute ICP elevations. Instead of NaCl, 0.25 to 1 g/kg bolus of Mannitol can also be given every 4 to 6 hours as needed  Recommended that patients receiving Mannitol should have their serum osmolality maintained below 320 mmol/L | If >20 mmHg for at least 5 minutes, then drain CSF  If ICP is still greater than 20 mmHg, administer bolus and/or infusion of hypertonic saline or mannitol.  Recommended that bolus of 3% hypertonic saline is given when there is intracranial hypertension, with the effective doses for acute use ranging between 2 and 5 mL/kg over 10 to 20 minutes | Use of Mannitol should be restricted to patients who, prior to ICP monitoring, have signs of transtentorial herniation or progressive neurological deterioration that is not due to extracranial causes  Use additional analgesia/sedation  Administer neuromuscular blockade, which should be monitored with EEG.  Administer additional hypertonic saline/hyperosmolar therapy  CT scan should be repeated to see if hemicraniectomy is necessary  As ICP improves along with clinical neurological recovery, then patient can be weaned off ICP, CCP, and/or PrO2 therapy |
| CPP Pathway | CPP of 60-70 mmHg. If the CPP is below optimal levels and if autoregulation is impaired, it is recommended for ICP to be lowered instead of elevating MAP.  Whether the minimal CPP should be 60 or 70 mmHg depends on the autoregulatory status.  Due to the risk of respiratory failure in adults, fluids and pressors can be used to maintain a CPP of 70 mmHg | CPP should be in between 40 to 50 mmHg in children who are five years old and under.  Those who are between 5 and 17 years old should have their CPP maintained above 50 mmHg | If CPP decreases, the status of the intravascular volume should be checked, and vasopressor infusion and hypertonic saline bolus should be given.  However, if CPP still decreases, then repeat a CT scan to see if surgery should be considered |
| PbrO2 Pathway |  |  | Brain tissue oxygen (PbrO2) should be kept greater than 10 mmHg if PbrO2 monitor is being used. Note that some literature suggests that PbrO2 less than 20 mmHg is considered compromised.  If PbrO2 drops, then raise FiO2  If PbrO2 still lowers, then give vasopressor infusion, adjust PaCO2, and optimize hemoglobin levels  Repeat a CT scan to see if surgery should be considered |
| Second Tier Therapies |  | Moderate hypothermia (between 32 and 34 degrees) and hyperventilation can also be considered  Avoid sodium concentrations greater than 160 mEq/L and osmolarity of greater than 360 mOsm/L. | If the CT scan reveals a new or growing swelling or hemorrhage, then, patient should undergo evacuation and DC.  Bifrontal DC is not suggested in patients who have diffuse injury (without having mass lesions) and patients who have ICP elevation of greater than 20 mmHg for more than 15 minutes within 1 hour that are refractory to first-tier therapies.  A large frontotemporoparietal DC is suggested over a small frontotemporoparietal DC.  Appropriate to administer dose of hypertonic saline or mannitol,  If hyperosmolar therapy and propofol infusion (titrated to deep sedation) does not lower ICP, then consider putting patient into barbiturate coma  Advanced neuromonitoring is also recommended, such as brain tissue oxygen monitoring to help guide bedside management of ICP. |
